# Clinical impact of prophylactic antibiotics in kidney transplantation: A retrospective observational cohort study with historical comparison

**DOI:** 10.1371/journal.pone.0337578

**Published:** 2025-11-21

**Authors:** Sang Ah Lee, Jin-Myung Kim, Hye Eun Kwon, Youngmin Ko, Joo Hee Jung, Sung Shin, Young Hoon Kim, Sung-Han Kim, Hyunwook Kwon

**Affiliations:** 1 Division of Vascular Surgery, Department of Surgery, Asan Medical Center, University of Ulsan College of Medicine, Seoul, Korea; 2 Division of Kidney and Pancreas Transplantation, Department of Surgery, Asan Medical Center, University of Ulsan College of Medicine, Seoul, Korea; 3 Department of Infectious Diseases, Asan Medical Center, University of Ulsan College of Medicine, Seoul, Korea; Medical University of Gdansk, POLAND

## Abstract

**Purpose:**

Optimal perioperative antibiotic prophylaxis in kidney transplantation remains undefined despite routine antibiotic administration to prevent infections. In this retrospective observational cohort study with historical comparison, we compared the clinical efficacy of 6 days of ampicillin/sulbactam vs. a single dose of cefazolin.

**Materials and methods:**

We retrospectively analyzed 2322 kidney transplantation recipients at a single center, with the evaluation period spanning from 2015 through 2021. Patients were divided into 2 groups based on the perioperative antibiotic regimen received: 971 patients received ampicillin/sulbactam, and 1351 received cefazolin. This study focused on evaluating the impact of these regimens on postoperative infection incidence and the 6-month acute rejection (AR) rates.

**Results:**

The cefazolin group exhibited a tendency toward higher urinary tract infection rates within 1 month after transplantation (3.4% vs. 2.2%, p= = 0.078). There were no significant differences in surgical site infections between the groups. The 6-month AR rates were significantly lower in the cefazolin group than in the ampicillin/sulbactam group (5.1% vs. 7.9%, p= = 0.009). Cefazolin was also confirmed to be significantly associated with reduced 6-month AR rates in the multivariable logistic regression analysis (odds ratio 0.63, 95% confidence interval [0.45-0.89], p= = 0.009).

**Conclusion:**

In this study, we observed that a single dose of cefazolin as perioperative antibiotic prophylaxis may lead to higher rates of postoperative urinary tract infections, but it could potentially lower the incidence of acute rejection within six months.

## Introduction

Kidney transplantation (KT), which requires immunosuppressive therapy, can lead to a range of infectious complications. Urinary tract infections (UTIs) commonly occur after KT and account for a significant proportion of hospitalizations post-transplantation. [1,2] Previous studies have shown that 7–80% of patients experience UTIs after KT, with the highest incidence occurring within the first 3 to 6 months [3,4]. Additionally, prophylactic antibiotics are routinely used in association with KT to help prevent surgical site infections (SSIs), similar to other abdominal surgeries and urological procedures [5]. Although occasional uncertainties over their effectiveness exist, the administration of prophylactic antibiotics is considered standard practice with KT. However, the specific regimens and durations of antibiotic administration vary significantly among transplant centers [5,6].

In KT, which is classified as a clean-contaminated surgical procedure, cefazolin and cefuroxime are predominantly used as prophylactic antibiotics [2,7,8]. These antibiotics are often administered as a single dose or for a short period. However, some centers use antibiotics targeting anaerobes and *Pseudomonas* spp. for extended periods to address a broader range of potential pathogens [8–10]. Nonetheless, minimizing the use of broad-spectrum antibiotics is recommended not only to reduce the emergence of multidrug-resistant bacteria but also to preserve gut microbiota equilibrium [11,12]. Several studies have indicated an interaction between the gut microbiome and immune homeostasis [13].

Given the established relationship between the use of broad-spectrum antibiotics and immune homeostasis, different preoperative antibiotic prophylaxis regimens may influence the rate of acute rejection (AR) following transplantation. Therefore, in this study, we compared the incidence of postoperative infection and AR rates among KT recipients treated with prophylactic antibiotics comprising 6 days of ampicillin/sulbactam vs. a single dose of cefazolin.

## Materials and methods

### Patients

This single-center, retrospective, observational study analyzed data from a consecutive cohort of patients who underwent KT at Asan Medical Center between January 2015 and December 2021. Five experienced surgeons performed the KT procedures. Among the 2661 patients who underwent KT during the study period, 239 were excluded for the following reasons: 62 had previously or simultaneously received a different organ transplant, 134 were taking therapeutic antibiotics during the surgery, and 43 were under the age of 18 years. Consequently, the study included a total of 2322 patients. The study protocol was approved by the Institutional Review Board of Asan Medical Center, Republic of Korea, which waived the requirement for informed patient consent because of the study’s retrospective design (Authorization code: 2022−1099). Data were accessed from August 13, 2022, to July 31, 2023, for research purposes. All data were fully anonymized prior to analysis to protect confidentiality. The investigation adhered to the ethical guidelines of the World Medical Association’s Declaration of Helsinki.

### Prophylactic antibiotics protocol

Patients received therapeutic antibiotics if they had positive urine culture results, including positive cultures from donor urine, or if they were being treated for any infection at the time of surgery. Those without indications for therapeutic antibiotics were administered prophylactic antibiotics immediately before the skin incision in the operating room. In February 2018, our center revised its prophylactic antibiotic protocol, transitioning from a 6-day regimen of ampicillin/sulbactam to a single dose of cefazolin. To evaluate the impact of this protocol change, we categorized the patients into 2 groups based on the timing of their transplantation relative to this policy update. In the ampicillin/sulbactam group, patients received ampicillin/sulbactam 2.25 g (composed of ampicillin 1.5 g and sulbactam 0.75 g) three times daily (TID), beginning with a preoperative dose administered prior to surgical incision, and continued through postoperative day (POD) 5. In the cefazolin group, patients received a single preoperative dose of cefazolin 2 g. For the prevention of pneumocystis pneumonia, patients who had not received rituximab prior to surgery were given daily trimethoprim-sulfamethoxazole for 6 months after transplantation. Those treated with rituximab before surgery were prescribed the medication for 1 year after transplantation [14].

### Surgical procedure

Transplant procedures adhered to standardized methods, including preoperative skin preparation with povidone–iodine and ethanol, followed by sterile draping. The primary technique for ureteral reconnection was the Lich-Gregoir approach, supplemented by occasional end-to-end anastomosis. A double-J stent was routinely placed and subsequently removed 1 month postoperatively. The abdominal wall closure involved a layered approach with Vicryl sutures, and a surgical drain was placed. The drain was removed when the outflow volume decreased to 50 mL or less.

### Desensitization and immunosuppression

Desensitization was performed for patients with ABO blood group incompatibility and positive flow cytometric crossmatch (FCXM) results. The desensitization protocol included a single dose of rituximab (100–500 mg) administered 2 weeks before plasmapheresis, with or without intravenous immunoglobulin. Induction therapy involved either basiliximab or antithymocyte globulin (ATG). During the initial postoperative period, the target trough levels were set at 7–10 ng/mL and 100–150 ng/mL for tacrolimus and cyclosporine, respectively. After the first postoperative year, these target concentrations were adjusted to 5–7 ng/mL for tacrolimus and 50–100 ng/mL for cyclosporine. Mycophenolate mofetil was initiated at a dosage of 1.5 g/day and typically maintained at this level for 1 year. Intraoperatively, 500 mg of methylprednisolone was administered, with gradual tapering to 5 mg twice daily over the first postoperative year.

### Microbiological methods

Clinical specimens, including urine, wound swabs, and drainage fluids, were inoculated onto solid media such as blood agar plate and MacConkey agar for aerobic bacterial culture. Cultures were incubated under appropriate atmospheric conditions and examined for bacterial growth. Culture results from drainage fluids (e.g., Jackson-Pratt drains) were interpreted with the possiblity of contamination in mind. Antimicrobial susceptibility testing was conducted using the disk diffusion method (Kirby-Bauer), adhering to the Clinical and Laboratory Standards Institute (CLSI) guidelines outlined in M100 [15]. Mueller-Hinton agar was employed for this purpose. For bacterial isolates not amenable to automated susceptibility testing systems—such as Gram-positive rods, *Haemophilus* spp., *Neisseria* spp., *Nocardia* spp., *Moraxella catarrhalis*, and *Vibrio* spp.—manual methods including disk diffusion or E-test (Epsilometer test) were utilized. Automated systems like MicroScan, VITEK, or Phoenix were used for other isolates, with manual methods employed as confirmatory tests when necessary.

### Definitions and outcomes

The primary outcomes were postoperative UTI and SSI rates after KT. For the accurate assessment of post-transplant infection rates, bacteriuria was initially identified by the presence of ≥100,000 CFU/mL of urinary pathogens in aseptically collected midstream urine samples. For precise measurement of infection rates after KT, we referred to the current guidelines, which distinguish symptomatic UTI from asymptomatic bacteriuria, but modified these definitions to create three categories for research purposes to better capture the clinical spectrum of post-transplant bacteriuria:

1. Classic UTI: The presence of bacteriuria with symptoms such as urinary frequency, urgency, dysuria, and suprapubic pain, or systemic signs like fever and pain around the transplant site
2. Treated bacteriuria (UTI*): When bacteriuria is detected and treated with antibiotics, regardless of urinary symptoms
3. Positive urinalysis (UTI**): Bacteriuria with either pyuria or the detection of nitrites in urinalysis.

SSIs were identified through clinical indicators, such as pain at the incision site accompanied by signs of infection, fluid accumulation at the surgical site with elevated inflammatory markers detected by imaging or laboratory tests, or inflammation at the drain insertion site, regardless of the presence of microorganisms in pus or bodily fluids from the surgical area. Infections included not only local signs of inflammation at the incision or drain insertion site, but also deeper infections such as fluid collections or abscesses detected on imaging. Subclassification of SSIs into superficial, deep incisional, or organ/space types was not performed due to the retrospective nature of the study and limited documentation in some cases.

Patients routinely underwent scheduled outpatient follow-up every 1–4 weeks for up to 3 months, during which urinalysis and clinical evaluations were performed. UTI and SSI events were identified retrospectively through chart review of outpatient and inpatient records. For the main analysis, the 1-month incidence of UTI and SSI was used as the primary outcome.

The secondary endpoint of our study was the effect of prophylactic antibiotics on AR rates following KT. To determine the influence of prophylactic antibiotic protocols on AR, we analyzed the occurrence of AR within the first 6 months post-KT, a period potentially impacted by prophylactic antibiotics. AR identification was determined by conducting a pathological evaluation under the Banff criteria [16]. In our standard practice, biopsies were conducted only when there were specific clinical indications rather than performing routine protocol biopsies.

### Statistical analysis

Categorical variables were calculated as frequencies or percentages and compared using the chi-square or Fisher’s exact test, as appropriate. Normally distributed continuous variables were calculated as means with standard deviations and compared using Student’s t-tests, whereas non-normally distributed continuous variables were calculated as medians with ranges and compared using the Mann-Whitney U test. Variables significantly associated with study outcomes were identified via logistic regression analysis, and the results were reported as odds ratios (ORs) with 95% confidence intervals (CIs). Variables with P-values below 0.1 in univariable analyses were included in the multivariable analysis. All statistical analyses were performed using SPSS Statistics for Windows, version 21.0 (IBM Corp., Armonk, NY, USA), with P-values <0.05 considered statistically significant.

## Results

### Patient demographics

During the study period, 2661 patients underwent KT at our center. The patients were divided based on the perioperative antibiotic regimens administered: ampicillin/sulbactam (n = 971) and cefazolin (n = 1351). In the analysis of baseline characteristics (**Table 1**), the average age (p < 0.001) and body mass index (p = 0.010) were higher in the cefazolin group than in the ampicillin/sulbactam group. The cefazolin group also had a higher proportion of living donors (p = 0.001) and showed a preponderance of tacrolimus over cyclosporine as the calcineurin inhibitor of choice (p < 0.001). Furthermore, a higher proportion of patients in the cefazolin group were identified as FCXM positive, indicating a higher immunological risk (p < 0.001), which was an indication for ATG induction therapy (p < 0.001).

**Table 1:** Baseline characteristics.

Variable	Total (n = 2322)	Ampicillin/Sulbactam (n = 971)	Cefazolin (n = 1351)	P-value
**Age, years**	49.6 ± 11.7	47.8 ± 11.3	50.8 ± 11.8	<0.001
**BMI, kg/m** ^**2**^	22.9 ± 3.7	22.8 ± 3.5	23.1 ± 3.7	0.010
**Male gender**	1336 (57.5)	546 (56.2)	790 (58.5)	0.28
**Donor type**				<0.001
Deceased	404 (17.4)	210 (21.6)	194 (14.4)	
Live	1918 (82.6)	761 (78.4)	1157 (85.6)	
**Cause of ESRD**				<0.001
Hypertension	344 (14.8)	138 (14.2)	206 (15.2)	
Diabetes mellitus	593 (25.5)	199 (20.5)	345 (29.2)	
GN	230 (9.9)	91 (9.4)	139 (10.3)	
IgA nephropathy	360 (15.5)	166 (17.1)	194 (14.4)	
FSGS	62 (2.7)	29 (3.0)	33 (2.4)	
PCKD	106 (4.6)	37 (3.8)	69 (5.1)	
Unknown	485 (20.9)	239 (24.6)	246 (18.2)	
Others	142 (6.1)	72 (7.4)	70 (5.2)	
**Dialysis type**				0.002
None	429 (18.5)	170 (17.5)	259 (19.2)	
HD	1697 (73.1)	695 (71.6)	1002 (74.2)	
CAPD	135 (5.8)	70 (7.2)	65 (4.8)	
HD+CAPD	61 (2.6)	36 (3.7)	25 (1.9)	
**Calcineurin inhibitor**				<0.001
Tacrolimus	2108 (90.8)	841 (86.6)	1267 (93.8)	
Cyclosporine	214 (9.2)	130 (13.4)	84 (6.2)	
**Induction**				<0.001
None	15 (0.6)	12 (1.2)	3 (0.2)	
Basiliximab	2013 (86.7)	878 (90.4)	1135 (84)	
ATG	294 (12.7)	81 (8.3)	213(15.8)	
**ABO incompatible**	577 (24.8)	224 (23.1)	353 (26.1)	0.09
**CDC positive**	39 (1.7)	13 (1.3)	26 (1.9)	0.27
**FCXM positive**	172 (8.2)	46 (5.4)	126 (10.1)	<0.001
**DSA MFI > 5000**	541 (23.3)	240 (24.7)	301 (22.3)	0.17
**HLA-mismatch**	3.2 ± 1.7	3.1 ± 1.7	3.3 ± 1.7	0.002
**PRA I**	17.3 ± 28.1	20.2 ± 29.5	15.2 ± 26.9	<0.001
**PRA II**	17.8 ± 28.6	17.7 ± 28.9	17.9 ± 28.3	0.86

Abbreviations: BMI, body mass index; ESRD, end-stage renal disease; GN, glomerulonephritis; FSGS, focal segmental glomerulosclerosis; PCKD, polycystic kidney disease; HD, hemodialysis; CAPD, continuous ambulatory peritoneal dialysis; ATG, anti-thymocyte globulin; CDC, complement-dependent cytotoxicity; FCXM, flow cytometry crossmatch; DSA, donor-specific antibodies; MFI, mean fluorescence intensity; HLA, human leukocyte antigen; PRA, panel reactive antibody.

Continuous data are presented as mean ± standard deviation. Categorical data are presented as number (%).

### Infection rate

**Fig 1:**
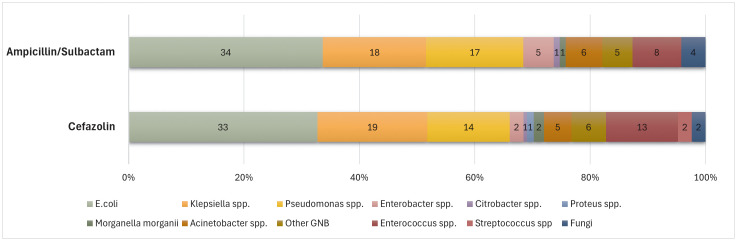
Identified microorganisms in urine within 1 month after kidney transplantation.

Compared with the ampicillin/sulbactam group, the cefazolin group had a significantly higher prevalence of UTI* (treated bacteriuria) (5% vs. 2.6%, p = 0.004). The rates of classic UTI and UTI** (positive urinalysis) were higher in the cefazolin group without statistical significance (UTI: 3.4% vs. 2.2%, p = 0.078; UTI**: 9.8% vs. 7.6%, p = 0.064). The prevalence of bacteriuria was higher in the cefazolin group (11.0%) than in the ampicillin/sulbactam group (8.8%), although the difference was not statistically significant (p = 0.082). There was no significant difference between the 2 groups in the rates of SSI, bacteremia, *Clostridium difficile* colitis, or multidrug-resistant infections (**Table 2**). In the multivariate analysis, male patients exhibited a significantly lower risk of UTIs than female patients (OR 0.28, 95% CI 0.16–0.48; p < 0.001). Moreover, the use of cefazolin as perioperative antibiotic prophylaxis was associated with an increased risk of UTIs relative to ampicillin/sulbactam (OR 1.72, 95% CI 1.02–2.93, p = 0.040) (Supplementary Table 1). The urinary microbiological profiles of the ampicillin/sulbactam and cefazolin groups were not significantly different from each other in terms of the distribution of pathogens. *Escherichia coli* was the predominant organism in both groups, followed by *Klebsiella* spp. *Enterococcus* spp., *Acinetobacter* spp., and *Pseudomonas* spp. were also detected among other species (**Fig 1**).

**Table 2:** Infection rates within 1 month after kidney transplantation.

Variable	Total (n = 2322)	Ampicillin/Sulbactam (n = 971)	Cefazolin (n = 1351)	P-value
**UTI**	67 (2.9)	21 (2.2)	46 (3.4)	0.078
UTI*	92 (4.0)	25 (2.6)	67 (5.0)	0.004
UTI**	207 (8.9)	74 (7.6)	133 (9.8)	0.064
**Bacteriuria**	233 (10)	85 (8.8)	148 (11.0)	0.082
**SSI**	37(1.6)	16 (1.6)	21 (1.6)	0.86
**Bacteremia**	44(1.9)	13 (1.3)	31 (2.3)	0.10
**UTI Septic shock**	2 (0.1)	0 (0)	2 (0.1)	0.51
***Clostridium difficile* colitis**	43 (1.9)	14 (1.4)	29 (2.1)	0.21
**MDR infection**	38 (1.6)	12 (1.2)	26 (1.9)	0.26

UTI: Bacteriuria plus symptoms such as urinary frequency/urgency, dysuria, suprapubic pain, or systemic symptoms, including fever and allograft pain.

UTI*: Bacteriuria treated with antibiotics.

UTI**: Bacteriuria associated with pyuria or the presence of nitrites in urinalysis.

Abbreviations: UTI, urinary tract infection; SSI, surgical site infection; MDR, multidrug-resistant.

Categorical data are presented as number (%).

### AR and mortality

Among all patients in both groups, the AR rate within 6 months post-transplantation was 6.3% (146/2322). During this period, the ampicillin/sulbactam group had an AR rate of 7.9%, while the cefazolin group had a rate of 5.1% (p = 0.006). No significant differences were observed in 1-year mortality and 1-year death-censored graft failure rates between the 2 groups (1-year mortality: 0.9% vs. 1.4%, p = 0.30; 1-year death-censored graft failure: 1.3% vs. 0.8%, p = 0.22) (**Table 3**). Even after adjusting for significant factors related to AR in the multivariate analysis, such as whether the donor was living or deceased (OR 0.23, 95% CI [0.15–0.36], p < 0.001), HLA-mismatch (OR 1.24, 95% CI [1.12–1.38], p < 0.001), and desensitization (OR 2.65, 95% CI [1.74–4.04], p < 0.001), the use of cefazolin remained associated with a lower incidence of AR compared with the use of ampicillin/sulbactam (OR 0.63, 95% CI [0.45–0.89], p = 0.009) (**Table 4**).

**Table 3:** Acute rejection rates and mortality rates.

Variable	Total (n = 2322)	Ampicillin/Sulbactam (n = 971)	Cefazolin (n = 1351)	P-value
**AR within 6 months**	146 (6.3)	77 (7.9)	69 (5.1)	0.006
**Mortality rate within** **1 year**	28 (1.2)	9 (0.9)	19 (1.4)	0.30
**DCGS within 1 year**	24 (1.0)	13 (1.3)	11 (0.8)	0.22

Abbreviations: AR, acute rejection; DCGF, death-censored graft failure.

Categorical data are presented as a number (%).

**Table 4:** Factors associated with the acute rejection within 6 months after transplantation.

	Univariable analysis		Multivariable analysis	
	OR (95 CI)	P–value	OR (95 CI)	P-value
**Female vs. male**	0.92 (0.65–1.28)	0.60		
**Living vs. deceased donor**	0.39 (0.27–0.55)	<0.001	0.23 (0.15–0.36)	<0.001
**Age (per 10 years)**	0.98 (0.85–1.13)	0.78		
**Body mass index (kg/m**^**2**^)	0.98 (0.94–1.02)	0.42		
**Cefazolin vs. ampicillin/sulbactam**	0.63 (0.45–0.87)	0.006	0.63 (0.45–0.89)	0.009
**Cyclosporine vs. tacrolimus**	1.23 (0.72–2.11)	0.45		
**HLA mismatch**	1.23 (1.11–1.34)	<0.001	1.24 (1.12–1.38)	<0.001
**Desensitization**	1.60 (1.14–2.26)	0.007	2.65 (1.74–4.04)	<0.001
**HD duration**	1.01 (1.00–1.01)	<0.001	1.0 (1.0–1.01)	0.33

Abbreviations: OR, odds ratio; HLA, human leukocyte antigen; HD, hemodialysis.

## Discussion

In our study, we found that the cefazolin group tended to have higher rates of UTIs within the first month post-transplantation, though this was not statistically significant, except for the UTI* (bacteriuria treated with antibiotics) subset. Furthermore, the occurrence of AR within the first 6 months after KT was lower in the cefazolin group compared to the ampicillin/sulbactam group. The incidence of SSIs was not significantly different between the 2 groups.

The documented prevalence of UTIs in KT recipients varies significantly, ranging from 1% to 40%, owing to discrepancies in diagnostic criteria and antibiotic prophylaxis. [2,6,17,18] In our study, symptomatic UTIs were documented at a rate of 2.9%, while bacteriuria associated with pyuria or the presence of nitrites in urinalysis (UTI**) was found in 8.9% of cases. The SSI rate of 1.6% in our study was comparable to, or lower than, the rates documented in recent studies, which have ranged from 2.4% to 11%. [5,18] Although the cefazolin group exhibited a higher incidence of symptomatic UTIs compared to the ampicillin/sulbactam group post-KT, this difference, as mentioned above, did not reach statistical significance. To adjust for differences in incidence resulting from varying definitions, we applied a modified classification into three categories (Classic UTI, UTI*, and UTI**) based on our predefined criteria, to comprehensively evaluate the spectrum of bacteriuria in post-KT patients. [2] Unlike symptomatic UTIs, the rate of UTI*, defined as bacteriuria treated with antibiotics, was significantly higher in the cefazolin group.

Upon reviewing previous studies, the Eurotransplant cohort study did not show significant differences in UTI rates between the single-dose cefazolin group and the multidose group, which covered gram-negative and anaerobic bacteria. [8] In a prospective, randomized controlled trial comparing single-dose versus multidose antibiotic prophylaxis protocols, UTI incidences were similar between groups. [19] However, that study excluded high-risk UTI groups like obese and diabetic patients and included patients in the single-dose group who were treated with cefotaxime, a gram-negative–covering antibiotic, marking differences from our research approach. [19] Research on the impact of prophylactic antibiotics on UTI incidence post-transplant has yielded inconsistent results across studies, with recent guidelines not providing strong evidence. Despite this, owing to the increasing risk of multidrug-resistant infections and lack of significant evidence supporting multidose prophylaxis in preventing post-transplant UTIs, current recommendations favor a single dose of first-generation cephalosporins. [2,20] However, in our study, the main pathogens associated with UTI were gram-negative bacteria. Additionally, KT recipients are at risk of maintaining indwelling catheters. Therefore, further research into alternative options, such as a single dose of second- or third-generation cephalosporins, which have limited activity against anaerobes and therefore may cause less disturbance to the gut microbiome balance, is warranted.

**Fig 2:**
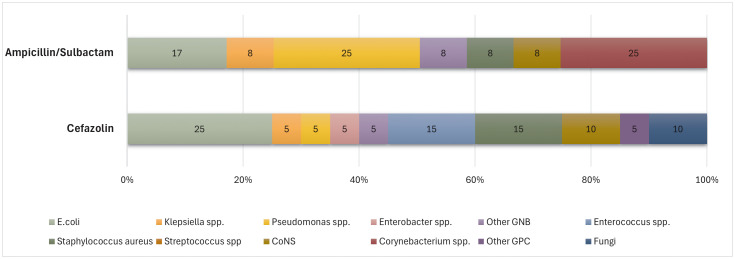
Identified microorganisms in the surgical site within 1 month after kidney transplantation.

The current guidelines recommend using a single dose of first-generation cephalosporins to prevent SSIs after KT. [5,21] The primary pathogens associated with SSIs in KT recipients are gram-positive bacteria, such as *Staphylococcus aureus*, coagulase-negative *staphylococci*, and *Enterococcus* spp. Recent evidence and guidelines suggest that for patients without risk factors, particularly in Asian cohorts like ours, a single dose of first-generation cephalosporins is effective in preventing SSI following surgery. [5,6,8] However, for recipients with risk factors—such as high body mass index, diabetes mellitus, surgical complications, or deceased donors—the use of broad-spectrum antibiotics has been shown to significantly contribute to the prevention of SSIs. [9] Additionally, bacterial cultures from SSI sites often identify not only gram-positive bacteria but also a notable presence of gram-negative bacteria, including *Klebsiella* spp., *E. coli*, and *Pseudomonas* spp. [5,9] In our study, among the 32 patients with SSIs from whom bacteria were cultured, *Klebsiella* spp. were found in 2 patients (6.25%), *E. coli* in 7 patients (21.88%), and *Pseudomonas* spp. in 4 patients (12.5%) (**Fig 2**).

In this study, patients who received a single dose of cefazolin had a lower rate of AR than those treated with a 5-day prophylactic course of ampicillin/sulbactam. The relationship between preoperative prophylactic antibiotic use and AR incidence remains underexplored, although recent research has mainly focused on how antibiotics affect immune responses through changes in gut microbiota. It is hypothesized that broad-spectrum antibiotics disrupt the gut’s microbial balance, leading to shifts in immune system homeostasis and promoting a proinflammatory response [13,22,23]. In a study using an aortic interposition model for vascular rejection in mice, it was found that disrupting the gut microbiota with antibiotics led to stronger immune attacks on transplanted arteries. This effect, characterized by a marked increase in T cells, macrophages, and neutrophils within the arterial walls, suggests an increased risk of AR [24].

In the field of KT, there remains a scarcity of clinical research on the impact of antibiotic use and its correlation with AR. In a study analyzing the gut microbiome of 26 KT recipients, 3 patients who experienced AR were treated with multiple antibiotics, and among these, 2 showed significant alterations in their gut microbiome, characterized by a decrease in Bacteroidetes and an increase in Lactobacillales [25]. This suggests a potential link between antibiotic-induced alterations in the gut microbiome and the development of anti-allograft immunity [25]. Although UTIs are generally considered a risk factor for AR in KT recipients, our study observed that the cefazolin group, despite a slightly higher incidence of UTIs, tended to have a significantly lower rate of AR. This finding may suggests that the immunological benefit associated with limited anaerobic coverage could outweigh the potential infection risk. Further studies are needed to clarify the mechanistic link between perioperative antibiotic selection, microbiome alteration, and allograft immune response.

A major strength of our study over previous research was its design as a single-center, retrospective observational cohort study with historical comparison and a large sample size of 2322 participants. This allowed for an in-depth analysis of the effects of prophylactic antibiotics on infection and rejection rates within a well-matched cohort.

Our study had several limitations. First, while our study included a consecutively enrolled cohort, the single-center, retrospective design may have introduced selection bias, including limitations in documentation and incomplete classification of surgical site infections, thereby limiting generalizability. Second, we did not directly validate the impact of antibiotics on AR through microbiome studies or animal experiments. Third, the study was conducted in an Asian population, and there may be racial or geographic variations in gut microbiota and antibiotic responses. Fourth, we did not maintain a consistent policy on asymptomatic bacteriuria after KT; however, defining UTI in 3 distinct ways allowed us to comprehensively analyze each aspect, effectively addressing potential biases associated with varying definitions of UTI.

## Conclusion

Our study highlighted the impact of prophylactic antibiotic regimens on UTI incidence and AR rates following KT. While the results showed that a single dose of cefazolin tended to increase UTI incidence, this was not statistically significant for symptomatic UTIs. Additionally, the findings suggest that cefazolin may potentially reduce AR rates than a 5-day course of ampicillin/sulbactam. Overall, cefazolin’s UTI prevention efficacy seems comparable to that of ampicillin/sulbactam, while its minimal impact on the gut microbiome may offer potential immunological benefits. Given the slight increase in UTI rates in the cefazolin group, further comparative studies with second-generation cephalosporins or quinolones, both of which also have a lesser impact on the gut microbiome while potentially reducing UTI occurrence, are warranted. Based on these findings, we preliminarily recommend considering narrow-spectrum, single-dose prophylactic antibiotics such as cefazolin as a preferred perioperative regimen in kidney transplantation, especially in standard-risk patients, to reduce the potential impact on gut microbiota and early immune activation.

## Supporting information

S1 Table Factors associated with UTI within 1 month after transplantation. (DOCX)

S2 File The complete raw dataset used for statistical analysis is provided as Supporting Information File S2 Raw data. (XLSX)

## References

[1] Cowan J, Bennett A, Fergusson N, McLean C, Mallick R, Cameron DW, et al. Incidence Rate of Post-Kidney Transplant Infection: A Retrospective Cohort Study Examining Infection Rates at a Large Canadian Multicenter Tertiary-Care Facility. Can J Kidney Health Dis. 2018;5:2054358118799692. doi: 10.1177/2054358118799692 30224973 PMC6136109

[2] Goldman JD, Julian K. Urinary tract infections in solid organ transplant recipients: Guidelines from the American Society of Transplantation Infectious Diseases Community of Practice. Clin Transplant. 2019;33(9):e13507. doi: 10.1111/ctr.13507 30793386

[3] Hollyer I, Ison MG. The challenge of urinary tract infections in renal transplant recipients. Transplant Infectious Dis. 2018;20(2). doi: 10.1111/tid.12828 29272071

[4] Parasuraman R, Julian K, AST Infectious Diseases Community of Practice. Urinary tract infections in solid organ transplantation. Am J Transplant. 2013;13 Suppl 4:327–36. doi: 10.1111/ajt.12124 23465025

[5] Abbo LM, Grossi PA, AST ID Community of Practice. Surgical site infections: Guidelines from the American Society of Transplantation Infectious Diseases Community of Practice. Clin Transplant. 2019;33(9):e13589. doi: 10.1111/ctr.13589 31077619

[6] Choi SU, Lee JH, Oh C-K, Shin GT, Kim H, Kim SJ, et al. Clinical significance of prophylactic antibiotics in renal transplantation. Transplant Proc. 2013;45(4):1392–5. doi: 10.1016/j.transproceed.2012.10.059 23726580

[7] Anesi JA, Blumberg EA, Abbo LM. Perioperative Antibiotic Prophylaxis to Prevent Surgical Site Infections in Solid Organ Transplantation. Transplantation. 2018;102(1):21–34. doi: 10.1097/TP.0000000000001848 28614192

[8] Bachmann F, Adam T, Friedersdorff F, Liefeldt L, Slowinski T, Budde K, et al. Perioperative antibiotic prophylaxis in renal transplantation: a single-center comparison between two regimens and a brief survey among the Eurotransplant renal transplantation centers. World J Urol. 2019;37(5):957–67. doi: 10.1007/s00345-018-2440-2 30109484

[9] Freire MP, Antonopoulos IM, Piovesan AC, Moura ML, de Paula FJ, Spadão F, et al. Amikacin prophylaxis and risk factors for surgical site infection after kidney transplantation. Transplantation. 2015;99(3):521–7. doi: 10.1097/TP.0000000000000381 25254907

[10] Sanclemente G, Bodro M, Cervera C, Linares L, Cofán F, Marco F, et al. Perioperative prophylaxis with ertapenem reduced infections caused by extended-spectrum betalactamase-producting Enterobacteriaceae after kidney transplantation. BMC Nephrol. 2019;20(1):274. doi: 10.1186/s12882-019-1461-4 31331289 PMC6647261

[11] Karam G, Chastre J, Wilcox MH, Vincent J-L. Antibiotic strategies in the era of multidrug resistance. Crit Care. 2016;20(1):136. doi: 10.1186/s13054-016-1320-7 27329228 PMC4916531

[12] Modi SR, Collins JJ, Relman DA. Antibiotics and the gut microbiota. J Clin Invest. 2014;124(10):4212–8. doi: 10.1172/JCI72333 25271726 PMC4191029

[13] Lee YK, Mazmanian SK. Has the microbiota played a critical role in the evolution of the adaptive immune system?. Science. 2010;330(6012):1768–73. doi: 10.1126/science.1195568 21205662 PMC3159383

[14] Kim YH, Kim JY, Kim DH, Ko Y, Choi JY, Shin S, et al. Pneumocystis pneumonia occurrence and prophylaxis duration in kidney transplant recipients according to perioperative treatment with rituximab. BMC Nephrol. 2020;21(1):93. doi: 10.1186/s12882-020-01750-8 32160881 PMC7066802

[15] Weinstein MP, Lewis JS. The Clinical and Laboratory Standards Institute Subcommittee on Antimicrobial Susceptibility Testing: Background, Organization, Functions, and Processes. J Clin Microbiol. 2020;58(3):e01864-19. doi: 10.1128/JCM.01864-19 31915289 PMC7041576

[16] Haas M, Sis B, Racusen LC, Solez K, Glotz D, Colvin RB, et al. Banff 2013 meeting report: inclusion of c4d-negative antibody-mediated rejection and antibody-associated arterial lesions. Am J Transplant. 2014;14(2):272–83. doi: 10.1111/ajt.12590 24472190

[17] Wojciechowski D, Chandran S. Effect of ciprofloxacin combined with sulfamethoxazole-trimethoprim prophylaxis on the incidence of urinary tract infections after kidney transplantation. Transplantation. 2013;96(4):400–5. doi: 10.1097/TP.0b013e3182962cab 23698597

[18] Ostaszewska A, Domagała P, Zawistowski M, Karpeta E, Wszoła M. Single-center experience with perioperative antibiotic prophylaxis and surgical site infections in kidney transplant recipients. BMC Infect Dis. 2022;22(1):199. doi: 10.1186/s12879-022-07182-z 35232378 PMC8886971

[19] Orlando G, Manzia TM, Sorge R, Iaria G, Angelico R, Sforza D, et al. One-shot versus multidose perioperative antibiotic prophylaxis after kidney transplantation: a randomized, controlled clinical trial. Surgery. 2015;157(1):104–10. doi: 10.1016/j.surg.2014.06.007 25304836

[20] Rodríguez Faba O, Boissier R, Budde K, Figueiredo A, Taylor CF, Hevia V, et al. European Association of Urology Guidelines on Renal Transplantation: Update 2018. Eur Urol Focus. 2018;4(2):208–15. doi: 10.1016/j.euf.2018.07.014 30033070

[21] Bratzler DW, Dellinger EP, Olsen KM, Perl TM, Auwaerter PG, Bolon MK, et al. Clinical practice guidelines for antimicrobial prophylaxis in surgery. Surg Infect (Larchmt). 2013;14(1):73–156. doi: 10.1089/sur.2013.9999 23461695

[22] Ubeda C, Pamer EG. Antibiotics, microbiota, and immune defense. Trends Immunol. 2012;33(9):459–66. doi: 10.1016/j.it.2012.05.003 22677185 PMC3427468

[23] Assar S, Nosratabadi R, Khorramdel Azad H, Masoumi J, Mohamadi M, Hassanshahi G. A Review of Immunomodulatory Effects of Fluoroquinolones. Immunol Invest. 2021;50(8):1007–26. doi: 10.1080/08820139.2020.1797778 32746743

[24] Rey K, Manku S, Enns W, Van Rossum T, Bushell K, Morin RD, et al. Disruption of the Gut Microbiota With Antibiotics Exacerbates Acute Vascular Rejection. Transplantation. 2018;102(7):1085–95. doi: 10.1097/tp.0000000000002169 29538261 PMC7228629

[25] Lee JR, Muthukumar T, Dadhania D, Toussaint NC, Ling L, Pamer E, et al. Gut microbial community structure and complications after kidney transplantation: a pilot study. Transplantation. 2014;98(7):697–705. doi: 10.1097/TP.0000000000000370 25289916 PMC4189837

