# Supplementary material for: Clinical impact of prophylactic antibiotics in kidney transplantation: A retrospective observational cohort study with historical comparison
Source: PLoS One. 2025 Nov 21;20(11):e0337578. doi: 10.1371/journal.pone.0337578 (PMC12637934; doi:10.1371/journal.pone.0337578)
Supplement: S1 Table — (DOCX) [file pone.0337578.s001.docx]

**Supplementary Table 1. Factors associated with UTI within 1 month after transplantation**

|  | **Univariable analysis** | | **Multivariable analysis** | |
| --- | --- | --- | --- | --- |
|  | **OR (95% CI)** | **P–value** | **OR (95% CI)** | **P-value** |
| **Male vs. female** | 0.28 (0.17–0.05) | <0.001 | 0.28 (0.16–0.48) | <0.001 |
| **Living vs. deceased donor** | 1.08 (0.56–2.07) | 0.83 |  |  |
| **Age (per 10 years)** | 1.08 (0.88–1.33) | 0.47 |  |  |
| **Diabetes mellitus** | 0.99 (0.58–1.69) | 0.97 |  |  |
| **Cefazolin vs. ampicillin/sulbactam** | 1.60 (0.95–2.69) | 0.08 | 1.72 (1.02–2.93) | 0.040 |
| **Cyclosporine vs. tacrolimus** | 1.15 (0.52–2.56) | 0.72 |  |  |
| **ATG vs. basiliximab** | 1.38 (0.72–2.68) | 0.33 |  |  |
| **HD duration** | 1.00 (1.00–1.01) | 0.08 | 1.0 (1.0–1.01) | 0.058 |
| **Desensitization** | 1.34 (0.81–2.21) | 0.26 |  |  |

Abbreviations: OR, odds ratio; ATG, antithymocyte globulin; HD, hemodialysis
